# No evidence for relationship between paternal post-partum depressive symptoms and testosterone or cortisol in first-time fathers

**DOI:** 10.3389/fpsyg.2024.1348031

**Published:** 2024-02-15

**Authors:** Daria A. Kotov, Randy Corpuz

**Affiliations:** Department of Psychology, University of Massachusetts Boston, Boston, MA, United States

**Keywords:** dual hormone hypothesis, testosterone, cortisol, paternal postpartum depression, fathers

## Abstract

Male life history strategies are regulated by the neuroendocrine system. Testosterone (T) and cortisol regulate male behaviors including parenting and facilitate managing tradeoffs at key transitions in development such as first-time fatherhood. Both hormones demonstrate marked fluctuations in the postnatal period, and this presents an opportunity to investigate the role of T and cortisol in postpartum depressive symptoms—comparably less studied in fathers than in mothers in the evolutionary literature. Prior work on depressive symptoms has yet to integrate insights from the “dual hormone hypothesis (DHH)” which has focused on how T and cortisol *interact* to jointly regulate traits associated with dominance and status-seeking (i.e., mating effort) but has yet to be included in models of parenting effort. In this research, we use secondary data to investigate the relationship between DHH and traits ostensibly opposed to status seeking (i.e., depressive symptoms). First-time fathers (*n* = 193) provided morning saliva samples 10 months following parturition and reported on the presence of depressive symptoms (BDI-II). Responses were decomposed into three factors: cognitive, affective, and somatic. Using hybrid latent variable structural equation modeling, we did not find evidence that T predicted variability in cognitive, affective, or somatic depressive symptom factors. We found a null effect for cortisol as well. Finally, we could not find evidence that the DHH variable (T × cortisol interaction) predicted any variability in cognitive, affective, or somatic depressive symptoms. While we did not find evidence to support our hypotheses using a secondary data set, this study contributes to research on the neuroendocrinology of depression in fathers. Discussion focuses on the limitations of sample demographics, timing of saliva and self-report collection, and the lack of extant theory specific to paternal postpartum depression.

## Introduction

Across species, male life history strategies are regulated by the neuroendocrine system (Bribiescas et al., 2012). Paternal care is exceedingly rare in mammals (Geary, 2007) and its expression is tied to testosterone (T)—a hormone produced by the hypothalamic pituitary gonadal (HPG) axis (Book et al., 2001; Gettler, 2020; see King and Hegadoren, 2002; Giannotti et al., 2022 for review). Cortisol—a hormone produced by the hypothalamic pituitary adrenal (HPA) axis—also appears to modulate paternal care (Kuo et al., 2018; see Sobral et al., 2022). Across the transition to parenthood, both hormones demonstrate marked fluctuations in expectant fathers and, most notably, in the postnatal period following the birth of offspring (Edelstein, 2022). For new fathers, the postnatal period and the cascade of endocrine ebbs and flows that accompanies this period of life represents a “critical window” for male health (Saxbe et al., 2018). Specific to postpartum depression—comparably less studied in fathers than in mothers—there exists mixed evidence as to the potentially harmful (Kim and Swain, 2007; Sundström Poromaa et al., 2017) or protective (e.g., Saxbe et al., 2017) factors of rapid endocrine shifts that are characteristic of fathers in the year following parturition.

In addition, the influence of cortisol and T on paternal postpartum depressive symptoms are frequently analyzed separately and have yet to integrate recent insights from the “dual hormone hypothesis (DHH)” literature (e.g., Knight et al., 2020) which has focused on how T and cortisol *interact* to jointly regulate male traits associated with dominance. According to the DHH, positive association between T and dominance traits (i.e., elevated status seeking) increased when cortisol levels are low (Popma et al., 2007; Mehta and Josephs, 2010; Mehta and Prasad, 2015; see Dekkers et al., 2019 for meta-analysis). These dominance-related traits are inversely related with conventionally understood symptoms of male depression where psychosocial motivation is decreased (Smith, 2013) and empathy is amplified (O’Connor et al., 2007; Vongas et al., 2018). To our knowledge, there are no studies looking at the potential relationship between the dual hormone hypothesis and specific phenotypes that include traits ostensibly opposed to status seeking (i.e., depressive symptoms).

### Testosterone and depression

There are sex differences between the role of T in men and women (McHenry et al., 2014). T decrease has been suggested to be correlated with depression especially in aging men (Schweiger et al., 1999; Seidman and Walsh, 1999; McIntyre et al., 2006). T supplementation in men has shown to reduce depressive symptoms (Walther et al., 2019). There have been some clinical trials of exogenous T administration supporting these correlations for men (Pope et al., 2003) however, this body of literature remains small.

Male T declines following the birth of offspring (see Gettler, 2020 for review). This decline is evident in other species where paternal care is relied upon (Wingfield et al., 2020) and is related to levels of parenting effort across cultures (e.g., Gettler et al., 2011; Corpuz et al., 2021; Beijers et al., 2022; see Grebe et al., 2019 for review). According to the Challenge Hypothesis (Wingfield et al., 1990), T declines following the arrival of offspring to facilitate a shift away from mating effort (e.g., behaviors tied to dominance, intra/intersexual competition, status-seeking) in favor of a behavioral suite that includes direct (e.g., feeding) and indirect (e.g., provisioning) parenting effort. It is possible that a moderate decline in T may be a species-typical reaction, but a more pronounced decrease may leave a new father susceptible to depressive symptoms. Indeed, Saxbe et al. (2017) found that new fathers with lower T expressed greater levels of depression.

Specific to postpartum depression in mothers, women with *higher* T experience greater levels of depressive symptoms (Rohr, 2002; Saxbe et al., 2017). High serum levels of T in women have been associated with depressive symptom cross culturally (Hohlagschwandtner et al., 2001; Aswathi et al., 2015; Trifu et al., 2019). Women with PPD have demonstrated higher levels of T measured 72 h following childbirth (Aswathi et al., 2015) and levels of T measured in the umbilical cord were higher in depressed mothers (Kikuchi et al., 2021). While this relationship between T and depression may be simple at first glance, more recent research reveals that a synchronous relationship of prenatal T between parents results in a higher drop of testosterone in fathers following birth and increased relationship quality reports (Cárdenas et al., 2023).

### Cortisol and depression

Findings on the relationship between cortisol and depression have been more complex compared to those of testosterone. A review of cortisol (sampled in morning and the afternoon) found that while basal and response cortisol levels appear the same between depressed and non-depressed individuals, depressed individuals experience blunted reactivity to stress and higher levels of cortisol in the recovery period (Burke et al., 2005). This summary of findings has been supported in subsequent studies (Fiksdal et al., 2019). Contrary to these findings, a study targeting the cortisol awakening response (CAR) found that individuals with acute depression had 25% more cortisol within the awakening curve, but those levels evened about an hour after waking up (Bhagwagar et al., 2005). More recently, sex differences have been observed in the cortisol responses and depression symptoms of couples with respect to relationship conflict. Depression in men was associated with higher cortisol levels while depression in women was related to a lower, flatter cortisol response curve (Powers et al., 2016).

Specific to new mothers, there are conflicting results as to the nature of the role of cortisol in maternal postpartum depression though the balance evidence suggests a negative relationship between cortisol and maternal PPD. A systematic literature review focusing on new mothers revealed that a blunted CAR was associated with cases of major maternal depression and that, while hypercortisolemia was linked to depressive states, hypocortisolemia was linked to chronic postpartum depression (Seth et al., 2016). Similarly, lower levels of cortisol predicted higher levels of maternal PPD in new mothers (Jahangard et al., 2019). There is also evidence that decreasing levels of cortisol starting in the second semester—a pattern opposite of an expected *rise* in cortisol as human mothers approach parturition—predicts maternal postpartum depression (Nierop et al., 2006; Caparros-Gonzalez et al., 2017). On the other hand, Gillespie et al. (2018) found a small *positive* relationships between cortisol and depressive symptoms in first-time mothers (see also Lang et al., 2021 for null relationship).

From a couple’s perspective, cortisol linkage between couples who were having their first child predicted fewer postpartum depressive symptoms for fathers (Khaled et al., 2021). Paternal postpartum depression is highly correlated with maternal postpartum depression (Kamalifard et al., 2018). However, the relationship between postnatal cortisol in fathers and their depressive symptoms has not been researched.

### Depression and dominance

Prior literature has found consistent *negative* relationships between T and traits ostensibly considered to be depressive symptoms. These include feeling tired (Määttänen et al., 2021), worthless (Ehrenreich et al., 1999), a diminished ability to focus (Booth et al., 1999) and reduced status-seeking motivation in low status men (Vermeer et al., 2020). Other literature has found *positive* associations between T and behaviors/traits such as extraversion (Smeets-Janssen et al., 2015), aggressive behavior (Carré et al., 2009, 2013), social dominance (Grant and France, 2001), and elevated sense of self (Eisenegger et al., 2017).

In some respect, cortisol acts the opposite way, with a *positive* relationship between cortisol and traits conventionally considered to be “depressive traits” such as loneliness (Doane and Adam, 2010). Most studies have not found any association of cortisol levels to traits like those above in healthy adults (Kirschbaum et al., 1992; Schommer et al., 1999; Munafo et al., 2006) nor in patients with chronic depression (Chopra et al., 2019; see Sundin et al., 2021).

### Current study

In this brief report, we have two goals: (1) add valuable data to the understudied topic of *paternal* postpartum depression and male endocrinology (T and cortisol); (2) explore the relationship between the dual hormone hypothesis and traits less typically studied in extant dual hormone literature (i.e., traits antagonistic to status seeking such as depressive symptoms). We use secondary data from a previously completed study on paternal care and T (see Corpuz et al., 2021 and/or Corpuz and Bugental, 2020). While this data was not originally collected to address either of the research goals stated above, this relatively large community sample of first-time fathers included cortisol and T assays in the postpartum period and measures of paternal postpartum depression. We view this project as a starting point to motivate future work that can further elucidate the relationships that we explore herein. Based on the balance of extant work, we hypothesize that depressive symptoms will be positively correlated with cortisol and negatively correlated with T. As our test of the DHH and depressive symptoms relationships is wholly novel, we heavily rely on our putative assumption that status-seeking traits are antagonistic to those nominally categorized as depressive symptoms—we predict that new fathers who are high in T and low in cortisol will demonstrate the lowest levels of depressive symptoms.

## Methods

### Overview and study design

The data used in this secondary analysis is from a single collection period drawn from a completed longitudinal study on paternal postpartum health outcomes for first-time fathers (see Corpuz and Bugental, 2020; Corpuz et al., 2021). Data on paternal depression and saliva swabs were collected approximately 10-months following childbirth (*M =* 289.85 days*, SD =* 24.95 days). All materials and procedures were reviewed and approved by the University of California Santa Barbara’s Institutional Review Board (IRB) where primary data collection took place while the corresponding author (RC) was previously in residence as a community researcher. Participants were provided information on the risks and benefits of participating in this research and signed consent forms prior to data collection. The data for this study was collected between 2013 and 2015 and all saliva assays (T and cortisol) were conducted between 2014 and 2015.

### Participants

For this study, *n* = 193 first-time fathers completed self-report measures and submitted saliva samples. Fathers were recruited from multiple sources: hospital birthing or community lactation classes (62.7% %), midwife referrals (15.7%), social media ads (13.6%), or community “Baby Basics” class (2.2%). The remaining 6% of the sample did not report a recruitment source. All participants were residing in Southern California (U.S.A.) at the time of data collection.

As noted in Corpuz and Bugental (2020), the average age of fathers in this study was *M =* 32.9, *SD* = 5.4, 84.1% of this sample was married to their child’s mother (at intake) and 77.4% of these fathers held at least a college degree. The median income of this sample was $50,000 to $75,000. Fathers self-reported their race/ethnicity as White (70.6%), Latino/Hispanic (12%), Asian American (5.2%), Black/African American (1.7%), Native American (1.3%), multiracial (2.6%), and other (3.9%). No differences were observed in study variables due to marital status (*p* = 0.79), household income (*p* = 0.61), or self-reported ethnicity (*p* = 0.68).

### Materials and procedure

Participants completed self-report questionnaires at the start of pre-planned home visits. Following the completion of self-report measures, home visitors trained fathers on how to expectorate saliva through a simulated collection procedure using the exact materials they would use on the morning of sampling. Parents were provided with pre-labeled saliva kits (sterile cotton swabs, polypropylene tubes, written instructions, and Ziploc bags) and a video demonstrating the process in detail.

#### Saliva collection

Fathers were instructed to expectorate saliva “within 30 min of waking up” during their next day off from work where applicable (i.e., a weekend day for most parents). This was an attempt to mitigate measuring neurohormonal fluctuations associated with physical activity and interpersonal stress that may be distinct from that usually experienced by new parents in the home environment (see Corpuz and Bugental, 2020). The specific day of sample selection (and subsequent sample retrieval) was agreed upon between the home visitor and the participant. Mean sampling times across participants was 6:47 am (*SD* = 1:09).

Fathers were told to abstain from alcohol (12 h prior), all food (1 h prior), and any beverages containing sugar, acid, or caffeine (5 min prior) leading up to their morning sample as per Granger et al. (2007). During sampling, fathers placed a sterilized absorbent cotton swab underneath their tongue for a minimum of 120 s. They then directed the swab into a polypropylene tube (using their tongue) and placed the tube into a freezer safe bag and into the freezer until samples were retrieved by a home visitor. Home visitors retrieved saliva samples from parents within 7 days of each visit.^1^

#### Saliva assays

Samples were assayed in duplicate at the Institute for Interdisciplinary Salivary Bioscience Research (IISBR; Arizona State University) using a highly sensitive competitive enzyme immunoassay (EIA)^2^ without modifications to the recommended protocols from Salimetrics (Carlsbad, CA).

##### Testosterone (T)

The test volume for T assay was 25 μL, and range of sensitivity was from 1.0 to 600 pg./mL. On average the inter- and intra-assay coefficients of variation were less than 15 and 10%, respectively. Reagents were stored at 2–8 degrees (C); reagents and samples were done without interruption across a 96-well microtiter plate coated with polyclonal anti-T antibodies. The full assay protocol can be downloaded directly from the manufacturer: https://salimetrics.com/wp-content/uploads/2018/03/testosterone-saliva-elisa-kit.pdf.

##### Cortisol

For cortisol, the assay range of sensitivity was 0.004–3.0 μg/dL. The detection limit was 0.018 μg/dL (after accounting for extraction dilution). On average the inter- and intra-assay coefficients of variation were less than 10 and 5%, respectively. Reagents were stored at 2–8 degrees (C); reagents and samples were done without interruption across a 96-well microtiter plate coated with monoclonal anti-Cortisol antibodies. The full assay protocol can be downloaded directly from the manufacturer: https://salimetrics.com/wp-content/uploads/2018/03/salivary-cortisol-elisa-kit.pdf.

### Covariates: T and cortisol

There were *n* = 27 fathers that self-reported smoking tobacco which can influence salivary assay values (see Granger et al., 2007). However, smoking status was not related to T (*p* = 0.77) nor cortisol (*p =* 0.21) in this sample of fathers. In this sample, neither cortisol nor T was related to a father’s age (*p*s > 0.34).

Father’s BMI was correlated with T (*p* = 0.08) and cortisol (*p* = 0.06)—though not statistically significant, BMI is included as a variable in all models. While the time of one’s morning sample was not related to T (*p* = 0.50), it was significantly related to a father’s level of morning cortisol (*p* = 0.002). As a result, we include time of day in subsequent models that include cortisol.^3^

After careful review of fathers who reported taking medications around the time of sampling, only one father reported taking a medication with well-documented effects on either cortisol or T production (i.e., aromatase inhibitor). Two other fathers self-reported taking psychiatric medications (i.e., Lamotrigine, Depakote) that, based on our reading of the literature, *may* influence T and/or cortisol though prior findings are mixed (e.g., Svalheim et al., 2009; Osório and Macedo, 2023). We elect for a conservative approach to handling these medications in our analyses; values for T and cortisol for all three fathers were removed and replaced as missing.^4^

#### Missing data and outliers: T and cortisol

In terms of other missing data, two fathers were missing data for both cortisol and T (one father provided insufficient quantity; one father’s sample had a concentration below lower limit of sensitivity). There were four fathers with outlying values (> 3SDs) for cortisol and one father with an outlying value for T. These cases are retained in analyses following maximum likelihood estimation to address missingness for cortisol and T.

#### Depressive symptoms

We used the Beck Depression Inventory (BDI-II; Beck et al., 1996) to measure depressive symptoms with this sample of first-time fathers. The BDI-II is a widely used self-report questionnaire in clinical and non-clinical community samples with high internal consistency and acceptable validity (García-Batista et al., 2018). Fathers use a Likert scale (0-not at all, 3-severely) to report on 21-items that assess mental and physical complaints related to depression such as loss of pleasure, self-criticalness, loss of energy, and tiredness/fatigue. Scores are summed across all 21 items to produce a single value representing one’s depression symptom score. In this sample, BDI-II scores were reliable (Cronbach α = 0.91). Overall raw sum scores (*M* = 7.92, *SD* = 6.84, range = 0 to 38) were in line with other community samples of fathers (e.g., Aviv et al., 2023).

As opposed to using sum/threshold scores in analyses, scores on the BDI II will be modeled as a three-factor model (cognitive, affective, somatic) to align with postpartum depression literature (Manian et al., 2013). Importantly, this three-factor modeling approach—compared to sum scoring the BDI II—also allows for more nuanced hypothesis testing concerning postpartum depression that is not possible when treating depression as a monolithic “single condition (where) all symptoms are interchangeable and equally good indicators” (Fried and Nesse, 2015).

## Results

Primary analyses for this brief report are executed using a structural equation modeling (SEM) framework which includes robust maximum likelihood estimation (MLE) missing data module in AMOS v.27 (IBM Chicago; Arbuckle, 2019). Separate models for T and cortisol—each predicting paternal depressive symptoms—were tested. A subsequent model to explore the dual hormone hypothesis will be built by creating a multiplicative interaction term (T × cortisol).

### Missing data

Overall, missingness for the variables tested in this study were moderate (0–3.1%) (Little and Rubin, 2019). To adjust for biases due to missing data, we fitted all models using the maximum likelihood estimation (MLE) missing data module in AMOS v.22. Data were missing completely at random (MCAR): Little’s MCAR test (*p* = 0.54).

### Three factor latent model

**Figure 1:**
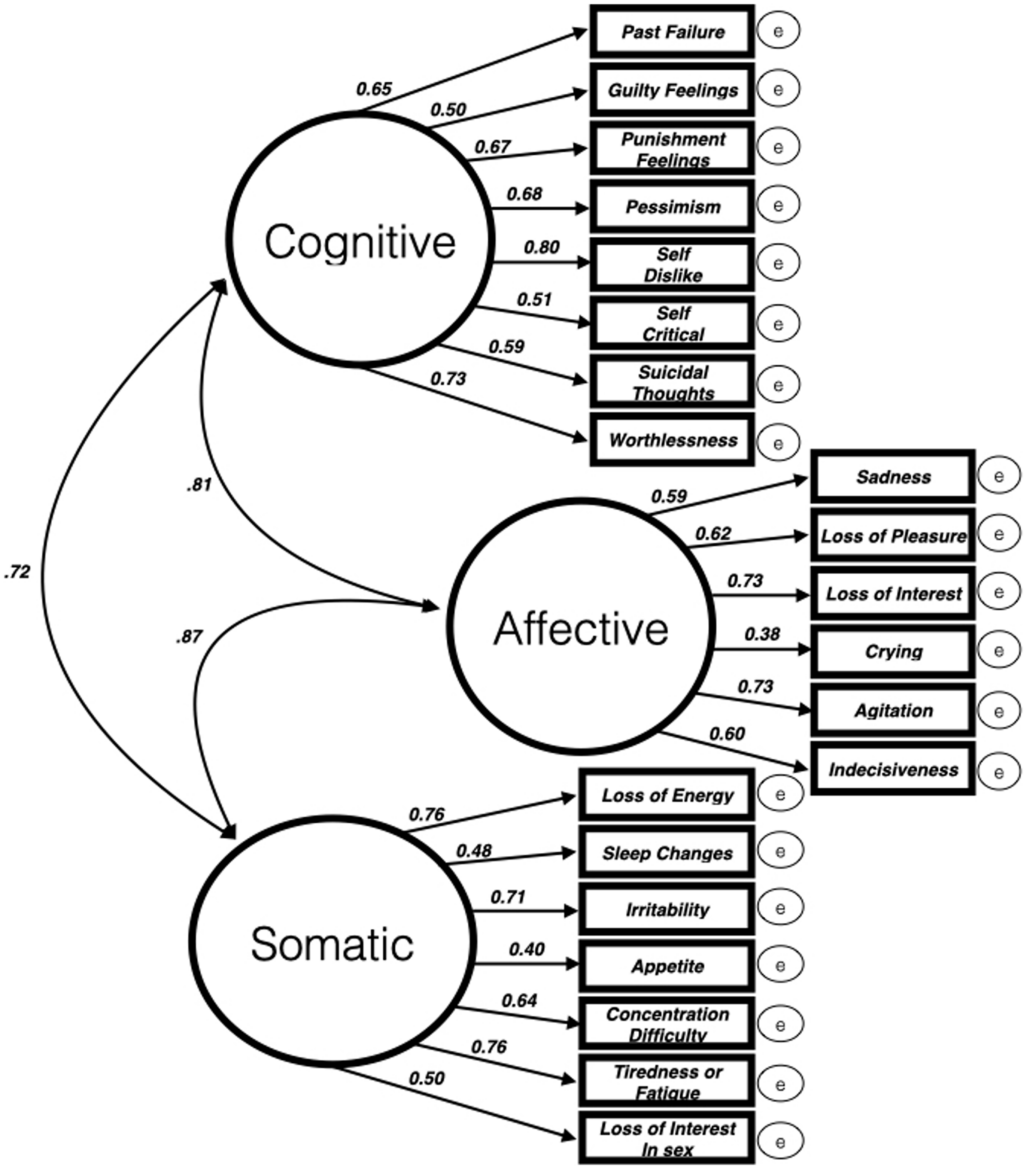
A three-factor latent model of BDI II depressive symptoms (Manian et al., 2013). All factor loadings are statistically significant (*p* < 0.001).

Using Manian et al. (2013) as a guide, we created a three-factor model of depressive symptoms. In this model, all 21 BDI II items are mapped onto three factors that, while highly correlated, represent theoretically distinct dimensions of depression. See Supplementary Table S1 for a full breakdown of each of the three factors (cognitive, affective, somatic), items that constitute each factor, and parameter and covariance estimates. All 21 loadings were significantly related to their higher order factor (all *p*s < 0.001) were retained in subsequent models. The three factor latent model is also depicted graphically on Figure 1.

### Hypothesis testing

Paternal T, while covaried with BMI, did not predict paternal depressive symptoms in our model. This null effect was true for T predicting cognitive (β = 0.05, *p* = 0.56), affective (β = 0.02, *p* = 0.77), and somatic (β = 0.03, *p* = 0.67) factors of depression. Full parameter estimates for this paternal T model appear on Supplementary Table S2.

We moved on to test a model whereby paternal cortisol—covarying for BMI and time of day—predicted a three-factor model for paternal depressive symptoms. In this second model, we also could not find evidence of a relationship between cortisol and paternal depressive symptoms whether they be cognitive (β = −0.02, *p* = 0.79), affective (β = −0.02, *p* = 0.78), or somatic (β = 0.01, *p* = 0.92). Full parameter estimates for this paternal cortisol model appear on Supplementary Table S3.

Contrary to some extant work on testosterone, cortisol, and paternal depressive symptoms, the current data suggests no such endocrine-depression relationships.

#### Dual hormone hypothesis

**Figure 2:**
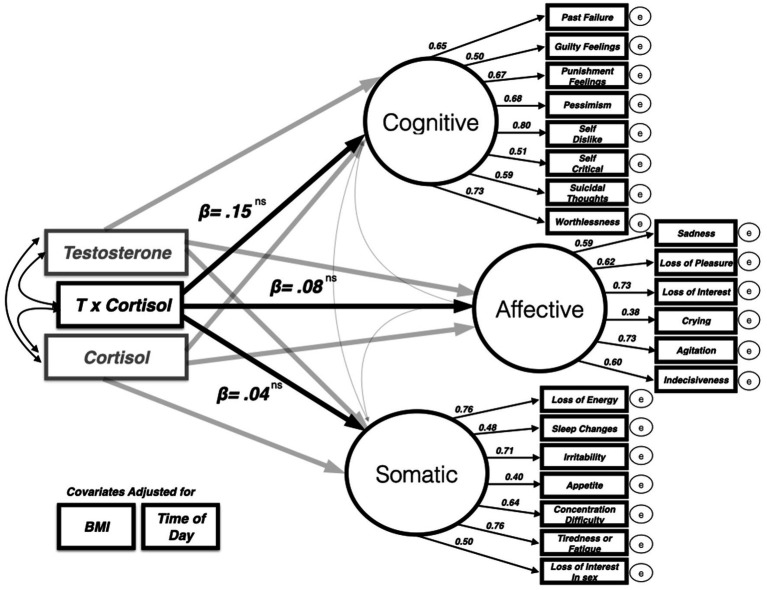
Dual hormone (testosterone × cortisol interaction) predicting three-factor latent model of BDI II depressive symptoms (only standardized beta values for dual hormone variable and each latent factor depicted for clarity). The model was a good fit for the data [CFI = 0.94, RMSEA = 0.048 (90% CI = 0.004–0.058)].

To explore the relationship between the dual-hormone hypothesis and paternal depressive symptoms, we built a hybrid latent variable structural equation model whereby the multiplicative T × cortisol interaction term predicted depressive symptoms while covarying for the following variables: T, cortisol, BMI, and time of day. The model was a good fit for the data [CFI = 0.94, RMSEA = 0.048 (90% CI = 0.004–0.058)]. A significant, negative interaction effect would be evidence for dual-hormone influence on depressive symptoms that could then be further probed using simple slopes analysis (see dual hormone meta-analysis from Dekkers et al., 2019). However, in this study, the dual hormone interaction variable could not predict any statistically significant variability in paternal depressive symptoms in this sample of fathers on any of the three depressive symptoms factors^5^ cognitive (β = 0.15, *p* = 0.63), affective (β = 0.08, *p* = 0.81), somatic (β = 0.04, *p* = 0.88). See Table 1 for full parameter estimates (also Figure 2).

**Table 1:** Parameter estimates for testosterone (T) × cortisol interaction and paternal depressive symptoms model.

	*Unstd.*	*SE*	*CR*	*p*	*Std.*
T × Cortisol → Cognitive	0.002	0.004	0.471	0.63	0.147
T × Cortisol → Affective	0.001	0.002	0.235	0.81	0.077
T × Cortisol → Somatic	0.001	0.004	0.142	0.88	0.044
Testosterone → Cognitive	0	0.002	−0.147	0.88	−0.026
Testosterone → Affective	0	0.001	−0.064	0.94	−0.012
Testosterone → Somatic	0	0.002	0.079	0.93	0.014
Cortisol → Cognitive	−0.267	0.542	−0.493	0.62	−0.119
Cortisol → Affective	−0.089	0.319	−0.277	0.78	−0.07
Cortisol → Somatic	−0.141	0.574	−0.246	0.80	−0.061
**Covariance**					
T × Cortisol ←→ Testosterone	980.40	130.9	7.489	***	0.654
T × Cortisol ←→ Cortisol	4.96	0.569	8.714	***	0.827
T × Cortisol ←→ BMI	−19.225	9.936	−1.935	0.05	−0.145
T × Cortisol ←→ Time of Day	−8.414	3.041	−2.766	**	−0.209
Testosterone ←→ Cortisol	1.602	0.614	2.61	**	0.194

*p < 0.05, **p < 0.01, ***p < 0.001.

## Discussion

In this brief research report, we used secondary data from a previously completed longitudinal study on a community sample of first-time fathers to examine the relationship between T, cortisol, and paternal postpartum depressive symptoms. We did not find evidence in any of our models that T or cortisol were related in any way to paternal postpartum depressive symptoms. The size of the effects we uncovered in our secondary data analysis were notably low. Our novel test of the DHH and paternal postpartum depressive symptoms also resulted in a null finding. We could not find evidence that the interaction between T and cortisol was related to paternal postpartum depressive symptoms.

Postpartum depression can last anywhere from three months up to two years with spontaneous recovery (American Psychiatric Association, 2013). Given that our saliva sample and self-report BDI-II was collected at about ten months postpartum, it is possible that we missed the peak onset of depressive symptoms within our sample of fathers. While prior research can readily detect maternal PPD at 12 months (or later) following childbirth (Quevedo et al., 2012; Mercier et al., 2013; see Goodman, 2004 for review), it remains an open question as to *when* paternal PPD may similarly persist and whether a “peak” time to detect PPD exists. Paulson and Bazemore (2010) found substantial heterogeneity in the timing of PPD in their meta-analysis but concluded that men experienced peak PPD symptoms between 2 and 6 months postpartum. Collecting data from a subsample of fathers around three-four months after birth (or measuring paternal PPD longitudinally starting earlier in the postnatal window) would have provided us with an opportunity to compare depressive symptoms and to measure differences in the potential relationship between paternal PPD and T or cortisol. It is also important for one to note that this community sample was not a clinical sample and, as often is the case with non-clinical studies on PPD (e.g., Aviv et al., 2023), overall presence of depressive symptoms is tempered.

### Limitations

#### Saliva sample

In 2019, a meta-analysis was published that argued that basal levels of cortisol do not support the dual hormone hypothesis (Dekkers et al., 2019). Similarly, other work has clarified that the acute cortisol response, as opposed to basal cortisol, is what is driving the dual hormone interaction (Prasad et al., 2019a,b). The study from which this data was used did not perform any cortisol response tasks such as the cold pressor task (Hines and Brown, 1932) so we used basal levels of cortisol. The saliva sample gathered in this study was collected from participants within 30 min of waking up. This does not account for the cortisol awakening response (CAR) when cortisol levels are increasing for the first 30–45 min after awakening (Clow et al., 2004). It is likely that the saliva sample we are using is a measurement that was taken somewhere along the awakening curve and therefore neither a basal level nor a response level. With regards to the CAR, the following protocol: Stalder et al. (2016) has been developed for deriving meaningful data from morning cortisol samples and should be adhered to in future studies involving cortisol measurements.

#### Measuring paternal postpartum depression

In the current study, we measured paternal PPD using the BDI-II (Beck et al., 1987)—a commonly used depression inventory in the study of PPD in mothers and fathers (e.g., Dudley et al., 2001). However, some have pointed out that this measure was designed primarily as a diagnostic tool and its validity with (non-clinical) community samples is suboptimal when compared to inventories such as McBride et al., 2014 Edinburgh Postpartum Depression Scale (EPDS; Su et al., 2007; Lai et al., 2010). In both instances, these inventories were created with clinical cutoff scores to help diagnose major depressive disorder and may lack the specificity to detect acute postpartum depression. For example, in the current sample, only 32 fathers (of *n* = 193) fathers would have met the minimum threshold for “mild depression” if we sum-scored the BDI-II and utilized it as a diagnostic tool. In the current study, we modeled depressive symptoms as a three-factor model as our goal was to measure variability in symptoms (Manian et al., 2013). However, our results could have been different if a valid and reliable measure of (specifically) paternal PPD were available.

There are sex differences in how depression manifests—while women demonstrate higher rates of diagnosis, men have higher incidence of self-harm ideation (Cuijpers et al., 2014; see also Cavanagh et al., 2017). Males (and, by extension, fathers) are also expected to occlude depressive symptoms on self-report inventories leading some to propose revised cutoff values and modified interpretation of scores on existing depression inventories (e.g., Matthey et al., 2001). Future research interested in the relationship between cortisol and/or T and depressive symptoms may consider emerging findings from literature on “paternal postpartum blues” where these sex differences in symptomology and reporting biases are more explicitly being considered in the creation of more tailored measure of depressive symptoms for fathers (Baldy et al., 2023).

#### Demographics

The participants of this study are closer to a WEIRD (white, educated, industrialized, rich, and democratic) sample rather than diverse. There is a wide range of PPD prevalence across samples of fathers and can be anywhere between ~6–30% (Randhawa et al., 2021; Mughal et al., 2022). A meta-analysis found that postpartum depression is common in about 8.75% of fathers cross-culturally (Rao et al., 2020). Western Pacific regions tend to have the highest rates of postpartum depression, followed by Americans, then Europeans. The economic and living conditions as well as poorer social support of the Western Pacific regions may be contributing to the higher prevalence rates. The current sample was mostly White, educated, and middle to high income.

#### Theory

Our predictions in this paper were atheoretical and based exclusively on extant findings. In mothers, there have been a few theories as to the evolutionary advantages of mild to moderate postpartum depression such as the Bargaining Model (Hagen, 2002). Different situations are associated with different manifestations of depression which may indicate the presence of context-specific adaptations that activate in response to local information related to resource availability or levels of threat and/or kin support (Keller and Nesse, 2006). However, this framework of thinking has only been applied to maternal postpartum depression. Due to inherent asymmetries in the costs and benefits of reproduction for males (Trivers, 1974) and the vast differences in human life histories between the sexes, it is unlikely that “depression” (postpartum or otherwise) is expressed indistinctly for mothers and fathers. More work is needed to develop, propose, and test theories that can be tailored to PPD in males.

### Future directions

Measuring the cortisol awakening response and reactive cortisol levels is something that our lab aims to do in future data collecting. Given the various roles that cortisol has in the body, having additional measures may clarify the inconsistencies that we are seeing across studies. Collecting salivary measures at 3–4 month postpartum and 5–6 months postpartum along with the 10-month collection timepoint will provide longitudinal data as to how much *change* we see in depression scores in the time following the birth. This is especially valuable for non-clinical samples that are taken from the community. Although Becks Depression Inventory is an often-used and validated measure, switching to the Edinburgh Postnatal Depression Scale (EPDS) would allow for a direct comparison to other postpartum results and have it be specifically catered to the symptoms of postpartum depression as opposed to other forms of more general depressive symptoms measured in the BDI-II (Cox et al., 1987). The spontaneous onset and recovery of depression in the postpartum period remains under researched. Having more measures of endocrine changes throughout this transition to fatherhood may elucidate the potential underlying biological mechanisms for depression and how it may dynamically change.

## Conclusion

While we did not find evidence to support our hypotheses using a secondary data set, this study contributes to the small collection of research on the neuroendocrinology of depression in fathers. Our null effects from this secondary data analysis on first-time fathers contribute to additional cumulative knowledge and provide information for researchers to estimate statistical power and anticipate/address roadblocks in design in their planning of further data collection and model building efforts (Munafò and Neill, 2016; Harms and Lakens, 2018). In testing the novel hypothesis related to the DHH, we proposed the idea that depressive symptoms may lie at the opposite end of a continuum that includes more dominant-like traits. In doing so, we hope to have also motivated further DHH-related thinking in domains outside of status-striving, leadership, and aggression.

## Data availability statement

The data analyzed in this study is subject to the following licenses/restrictions: the data that support the findings of this study are available from the corresponding author RC, upon reasonable request. Requests to access these datasets should be directed to RC, randy.corpuz@umb.edu.

## Ethics statement

The studies involving humans were approved by University of California Santa Barbara IRB. The studies were conducted in accordance with the local legislation and institutional requirements. The participants provided their written informed consent to participate in this study.

## Author contributions

DK: Conceptualization, Data curation, Formal analysis, Investigation, Methodology, Software, Visualization, Writing – original draft, Writing – review & editing. RC: Conceptualization, Data curation, Formal analysis, Funding acquisition, Investigation, Methodology, Project administration, Resources, Software, Supervision, Validation, Visualization, Writing – original draft, Writing – review & editing.

## Footnotes

^1^78.4% of all samples retrieved were collected within seven days of participant expectorating saliva. There were no differences on study variables between samples picked up within 7 days or samples retrieved after 8+ days (all *ps* > 0.41).

^2^EIA’s correlation with tandem mass spectrometry—which has emerged as a preferred option for many labs in the years since assays were conducted for this study—is unstable (Welker et al., 2016; Prasad et al., 2019a,b) and there is ongoing debate as to the utility of using one method over the other (e.g., Mazur and Clifton, 2018). Because we did not utilize tandem mass spectrometry in the current study, our ability to contribute to this debate is limited.

^3^Data were not available on time *since* wake and, as a result, we do not have data to assess awakening responses for cortisol (Fries et al., 2009) or T (i.e., Kuzawa et al., 2016). See Limitations below.

^4^Most other medications listed by fathers in this sample were for allergies (e.g., OTC nasal spray, decongestants), pain (e.g., ibuprofen), or digestive issues (e.g., acid reflux).

^5^In a follow up analysis using the sum score of the BDI-II, null results were identical. The dual hormone interaction did not predict BDI-II sum score (β= 0.04, *p* = 0.62). As an additional follow up analysis, we created a first-order latent variable with parcels for cognitive (8 items), affective (6 items), and somatic (7 items) subscales. The dual hormome interaction did not predict variability in this latent variable with three parcels (β= 0.03, *p* = 0.68).
