# Supplementary material for: No evidence for relationship between paternal post-partum depressive symptoms and testosterone or cortisol in first-time fathers
Source: Front Psychol. 2024 Feb 15;15:1348031. doi: 10.3389/fpsyg.2024.1348031 (PMC10902172; doi:10.3389/fpsyg.2024.1348031)
Supplement: Supplementary file 1 [file Data_Sheet_1.docx]

**Supplementary Table 1.** Parameter estimates and critical ratios for the three-factor model of paternal depressive symptoms measured using the BDI II.

| **Parameters** | ***Unstd.*** | ***SE*** | ***CR*** | ***p*** | ***Std.*** |
| --- | --- | --- | --- | --- | --- |
| ***Cognitive*** | |  |  |  |  |
| V1 (Past Failure) | 1.00 | *nt* | *nt* | *nt* | 0.65 |
| V2 (Guilty Feelings) | 0.652 | 0.106 | 6.152 | *** | 0.498 |
| V3 (Punishment Feelings) | 0.731 | 0.092 | 7.912 | *** | 0.665 |
| V4 (Pessimism) | 0.855 | 0.106 | 8.067 | *** | 0.68 |
| V5 (Self-Dislike) | 1.143 | 0.125 | 9.156 | *** | 0.80 |
| V6 (Self-Critical) | 0.802 | 0.128 | 6.278 | *** | 0.509 |
| V7 (Suicidal Thoughts) | 0.455 | 0.063 | 7.182 | *** | 0.593 |
| V8 (Worthlessness) | 0.785 | 0.092 | 8.556 | *** | 0.732 |
| ***Affective*** | |  |  |  |  |
| V9 (Sadness) | 1.00 | *nt* | *nt* | *nt* | 0.591 |
| V10 (Loss of Pleasure) | 1.622 | 0.236 | 6.867 | *** | 0.62 |
| V11 (Loss of Interest) | 1.903 | 0.249 | 7.648 | *** | 0.725 |
| V12 (Crying) | 0.626 | 0.136 | 4.619 | *** | 0.381 |
| V13 (Agitation) | 1.73 | 0.225 | 7.691 | *** | 0.731 |
| V14 (Indecisiveness) | 1.459 | 0.218 | 6.684 | *** | 0.598 |
| ***Somatic*** | |  |  |  |  |
| V15 (Loss of Energy) | 1.00 | *nt* | *nt* | *nt* | 0.762 |
| V16 (Sleep Changes) | 0.843 | 0.132 | 6.366 | *** | 0.481 |
| V17 (Irritability) | 1.001 | 0.104 | 9.599 | *** | 0.709 |
| V18 (Appetite) | 0.627 | 0.119 | 5.288 | *** | 0.403 |
| V19 (Concentration) | 0.81 | 0.095 | 8.549 | *** | 0.636 |
| V20 (Tiredness/Fatigue) | 1.064 | 0.103 | 10.366 | *** | 0.762 |
| V21 (Lost Interest in Sex) | 0.569 | 0.087 | 6.574 | *** | 0.503 |
| **Covariance** |  |  |  |  |  |
| Cognitive 🡨🡪 Affective | 0.076 | 0.014 | 5.435 | *** | 0.816 |
| Somatic 🡨🡪 Affective | 0.085 | 0.014 | 5.887 | *** | 0.868 |
| Somatic 🡨🡪 Cognitive | 0.123 | 0.021 | 5.785 | *** | 0.72 |

*Note*. “nt” = not tested (parameter fixed to set scale for latent indicators); * *p* < .05, ** *p* < .01, *** *p* < .001.

**Supplementary Table 2.** Parameter estimates for testosterone and paternal depressive symptoms model.

| **Parameters** | ***Unstd.*** | ***SE*** | ***CR*** | ***p*** | ***Std.*** |
| --- | --- | --- | --- | --- | --- |
| Testosterone 🡪 Cognitive | 0 | 0.001 | 0.582 | 0.56 | 0.046 |
| Testosterone 🡪 Affective | 0 | 0 | 0.291 | 0.77 | 0.024 |
| Testosterone 🡪 Somatic | 0 | 0.001 | 0.42 | 0.67 | 0.034 |
| **Covariance** |  |  |  |  |  |
| Testosterone 🡨🡪 BMI | -22.415 | 13.61 | -1.65 | 0.1 | -0.12 |
| BMI 🡪 Cognitive | 0.015 | 0.008 | 1.83 | 0.06 | 0.146 |
| BMI 🡪 Affective | 0 | 0.005 | 0.015 | 0.98 | 0.001 |
| BMI 🡪 Somatic | 0.01 | 0.008 | 1.209 | 0.22 | 0.097 |

*Note:* * *p* < .05, ** *p* < .01, *** *p* < .001.

**Supplementary Table 3.** Parameter estimates for cortisol and paternal depressive symptoms model.

|  | ***Unstd.*** | ***SE*** | ***CR*** | ***p*** | ***Std.*** |
| --- | --- | --- | --- | --- | --- |
| Cortisol 🡪 Cognitive | -0.046 | 0.177 | -0.259 | 0.79 | -0.02 |
| Cortisol 🡪 Affective | -0.029 | 0.104 | -0.275 | 0.78 | -0.023 |
| Cortisol 🡪 Somatic | -0.018 | 0.187 | -0.099 | 0.92 | -0.008 |
| **Covariance** |  |  |  |  |  |
| Cortisol 🡨🡪 BMI | -0.108 | 0.055 | -1.967 | 0.05 | -0.148 |
| Cortisol 🡨🡪 Time of Day | -0.057 | 0.017 | -3.367 | *** | -0.257 |
| Time of Day 🡨🡪 BMI | 0.579 | 0.366 | 1.582 | 0.11 | 0.118 |
| BMI 🡪 Cognitive | 0.014 | 0.008 | 1.674 | 0.09 | 0.134 |
| BMI 🡪 Affective | -0.001 | 0.005 | -0.132 | 0.89 | -0.011 |
| BMI 🡪 Somatic | 0.01 | 0.009 | 1.214 | 0.22 | 0.098 |
| Time of Day 🡪 Cognitive | 0.023 | 0.027 | 0.833 | 0.40 | 0.068 |
| Time of Day 🡪 Affective | 0.01 | 0.016 | 0.635 | 0.52 | 0.054 |
| Time of Day 🡪 Somatic | -0.017 | 0.029 | -0.582 | 0.56 | -0.048 |

*Note:* * *p* < .05, ** *p* < .01, *** *p* < .001.
